# Cervical angioleiomyoma: A rare tumor behind persistent menorrhagia and pelvic pain

**DOI:** 10.18632/oncoscience.659

**Published:** 2026-05-22

**Authors:** Ipsita Mohapatra, Sarojini Raman, Subha Ranjan Samantaray, Mukul Panda

**Affiliations:** ^1^Department of Obstetrics and Gynaecology, All India Institute of Medical sciences, Bhubaneswar, India; ^2^Department of Pathology and Laboratory Medicine, All India Institute of Medical sciences, Bhubaneswar, India; ^3^Department of Obstetrics and Gynaecology, SUM Campus 2, Bhubaneswar, India

**Keywords:** angioleiomyoma, cervix, soft tissue tumor, menorrhagia, pelvic pain

## Abstract

Introduction: Angioleiomyoma is a rare, benign soft tissue tumor derived from smooth muscle cells with prominent vascular components. While more commonly found in the extremities, angioleiomyomas of the female genital tract, particularly the cervix, are extremely rare. Ultrasound helps in the diagnosis, but MRI is more definitive, as the vascular component of these tumors can often be highlighted with hyper intensity on T2-weighted images. Final confirmation of angioleiomyoma is by histopathological and immunohistochemistry studies.

Case report: We present here the diagnosis and management by total excision of a case of a large sized cervical angioleiomyoma in a young female, which presented as persistent heavy menstrual bleeding and pelvic pain.

Conclusions: Cervical angioleiomyoma, though rare, should be considered in the differential diagnosis of young females presenting with persistent heavy menstrual bleeding and pelvic pain.

## INTRODUCTION

Angioleiomyoma is an uncommonly encountered, benign soft tissue tumor of smooth muscle cell origin and increased vasculature. These constitute 4.5% of all benign smooth muscle tumors [1]. Though more frequently seen in the extremities, occurrence in female genital tract, particularly the cervix, have been least reported till date in literature. Clinically these tumors present with abnormal uterine bleeding and pelvic pain, often mimicking more commoner other gynecological conditions. We present a case of a large cervical angioleiomyoma in a young female, which presented as persistent heavy menstrual bleeding and pelvic pain.

## CASE PRESENTATION

A 26-year-old unmarried female presented to the gynecology outdoor with complaints of irregular menstrual cycles, chronic heavy menstrual bleeding and intermittent pelvic pain over the past three to four months. Her menstrual cycles had progressively increased in duration, accompanied by heavy bleeding, requiring multiple changes of sanitary pads each day. The patient also experienced cramping, non-radiating pelvic pain that worsened during menstruation.

The patient had no significant medical or surgical history. Routine blood investigations showed moderate anemia with hemoglobin value 8 g/dL consistent with the patient’s prolonged bleeding. Per abdomen examination was normal with no significant findings. Pelvic examination revealed a polypoidal mass of about 6 × 5 cm distending the cervix and cervical rim could be felt all around the mass. The pedicle of the mass could not be reached. A transvaginal ultrasound was performed which revealed a large mixed echogenic space occupying lesion most likely cervical fibroid or polyp of size 5.9 × 5.6 cm posterior to the cervical canal.

MRI of the pelvis was planned which confirmed the presence of a well-demarcated, hyper intense lesion on T2-weighted images, located within the cervix. The imaging characteristics were suggestive of a vascular component, consistent with a possible angioleiomyoma.

**Figure 1:**
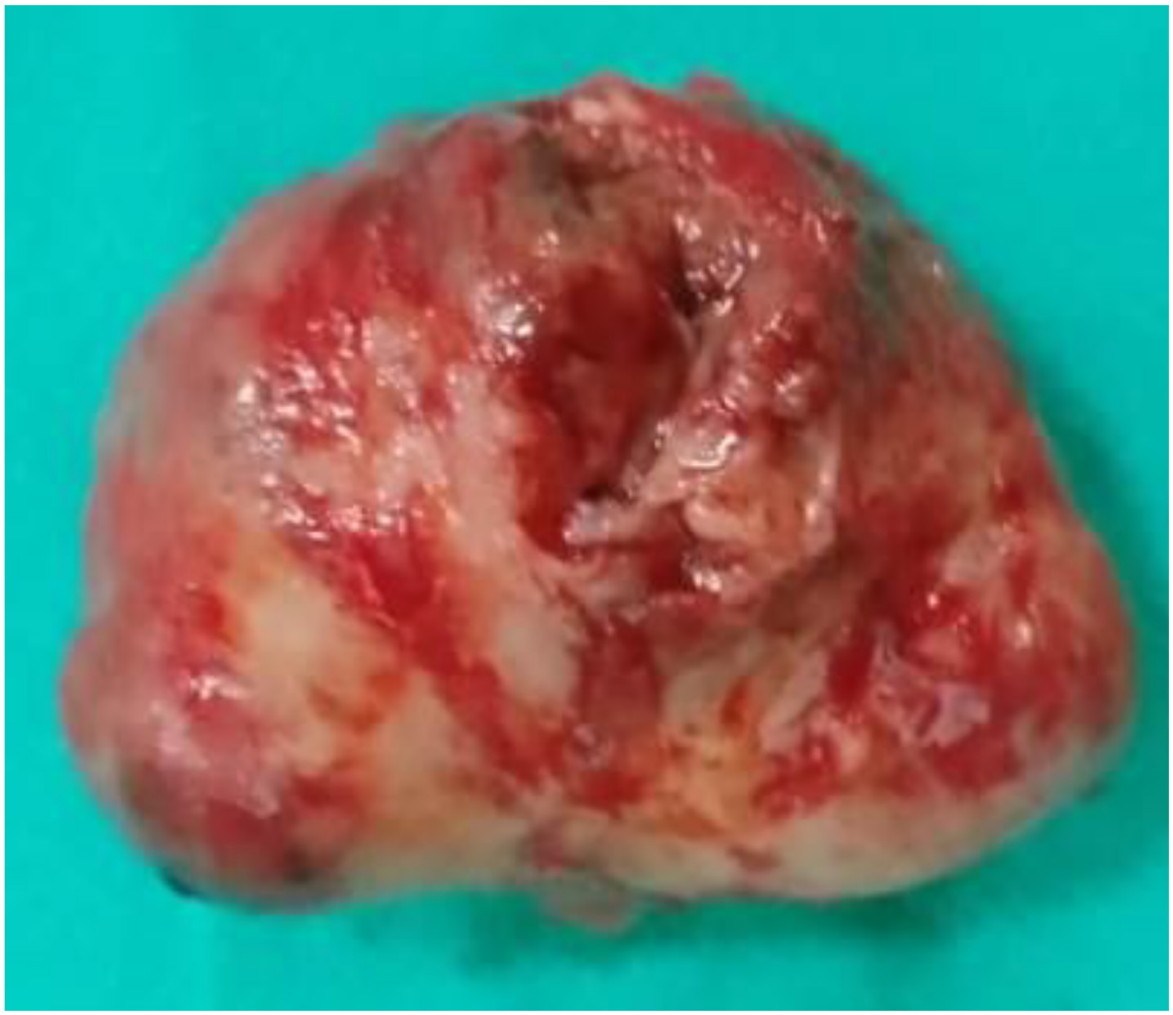
Gross appearance of the tumor.

The decision was made to proceed with a total excision of the tumor via a vaginal approach. The surgery was uneventful, and the tumor was completely resected. Hemostasis was ensured and the raw areas were coagulated. The tumor was irregularly round solid mass with prominent vascularity on its surface (Figure 1).

The post operative period was uneventful and the patient was discharged on the third post operative day.

**Figure 2:**
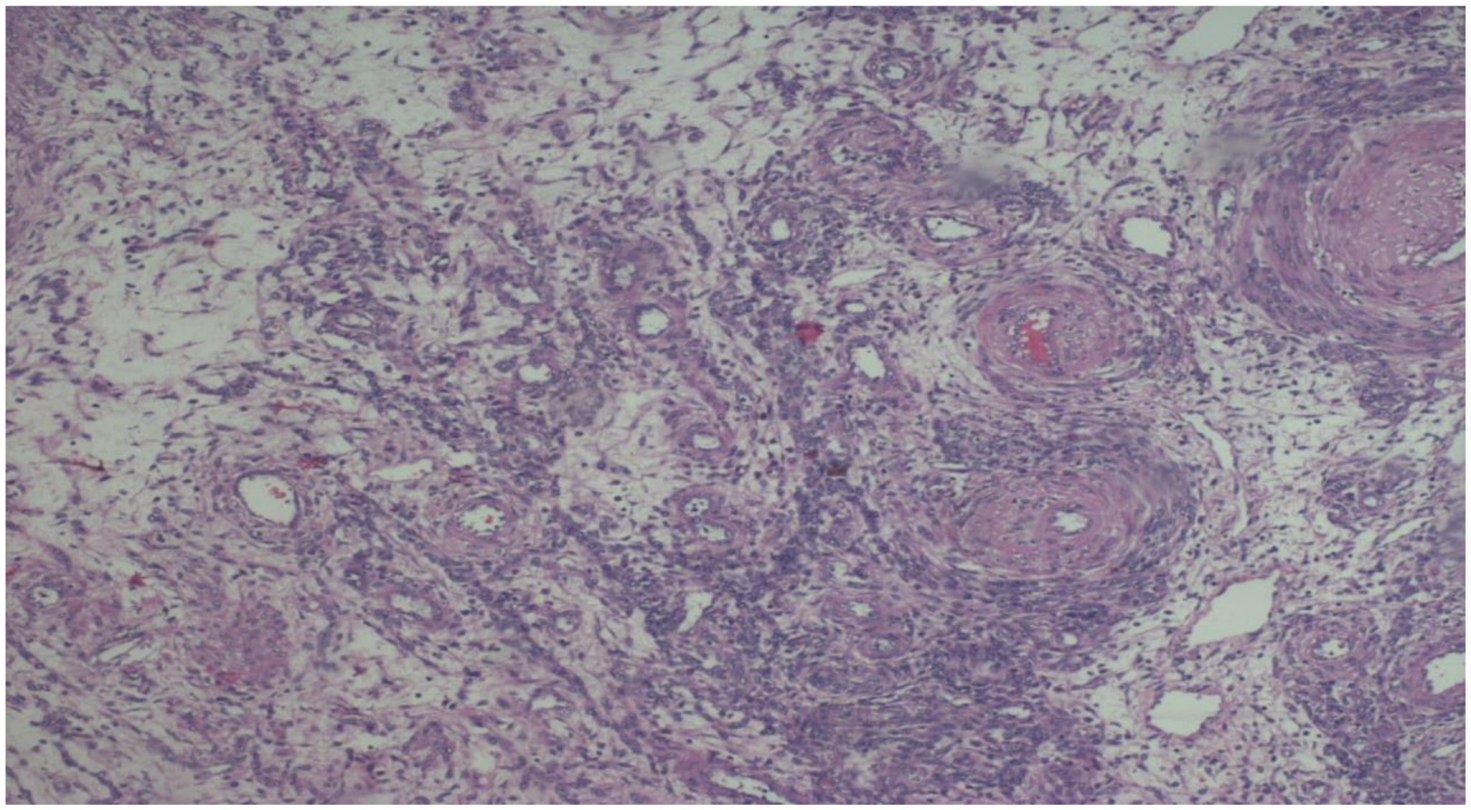
HE section shows perivascular accentuation of spindle shaped smooth muscle cells with surrounding myxoid stroma (10X).

Histopathological examination of the sections from the mass revealed a cellular spindle cell neoplasm arranged in long and short fascicles intersecting each other at right angles, concentrically around thick-walled blood vessels with foci of prominent myxoid stroma and cystic changes (Figure 2). There was no evidence of malignancy.

**Figure 3:**
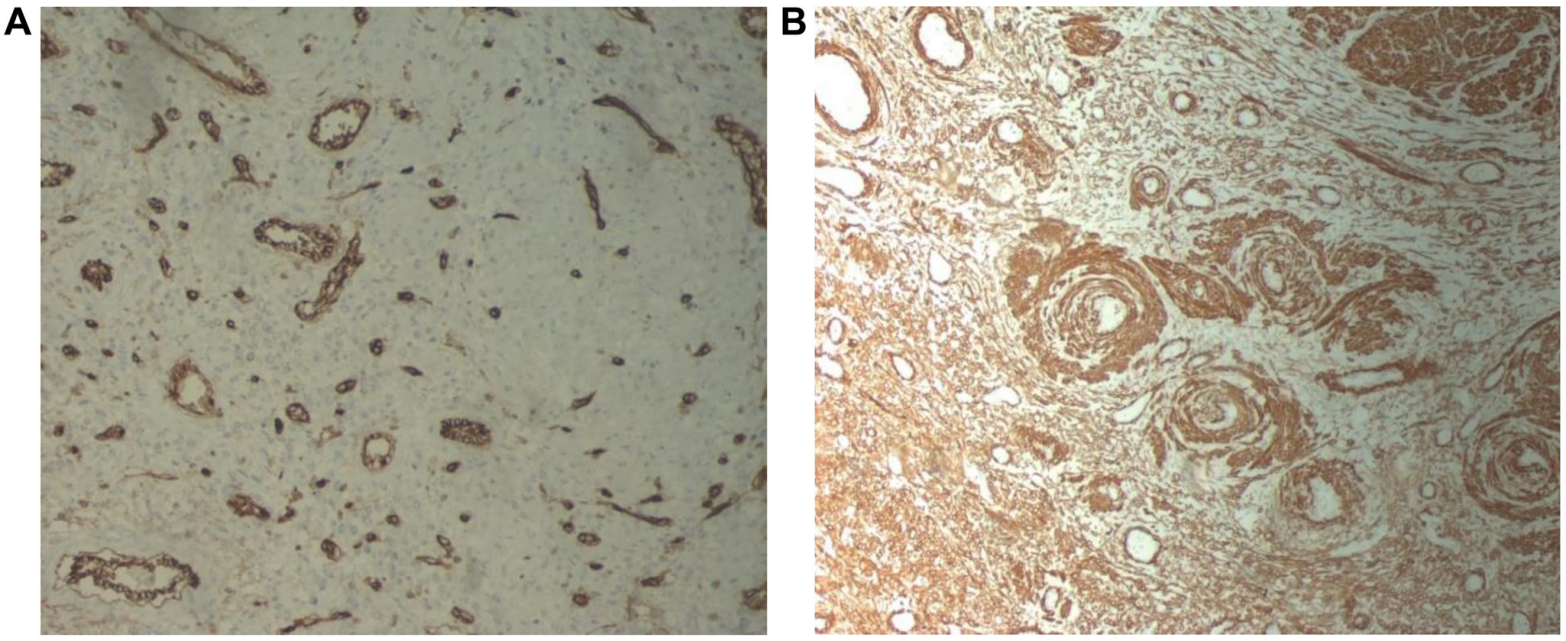
(**A**) Immunohistochemistry of angioleiomyoma positive for Smooth muscle antigen (10X). (**B**) Positive CD34 staining (20X).

Immunohistochemistry was positive for smooth muscle antigen (SMA) and CD34, confirming the diagnosis of a benign angioleiomyoma (Figure 3A, 3B).

At her 3-month follow-up, the patient reported complete resolution of her heavy menstrual bleeding and pelvic pain.

## DISCUSSION AND REVIEW OF LITERATURE

### Epidemiology

Angioleiomyomas are rare benign tumors of mesenchymal origin. While leiomyomas are the most common benign uterine tumors, their angioleiomyomatous variant is much less frequently observed in the uterus, cervix, broad ligament and any other part of the reproductive tract [2, 3]. About 0.3–0.4% of leiomyomas are angioleiomyomas [4]. The occurrence of angioleiomyoma in the female genital tract is exceedingly rare, with published reports limited to 15 cases in the uterine corpus and 6 cases in the cervix [5, 6]. According to World Health Organization classification of soft tissue tumors in 2020, angioleiomyomas belong to the group of perivascular tumors [7]. Pathogenesis of angioleiomyomas is not clear. Angioleiomyomas arise from perivascular smooth muscle cells possibly due to some endothelial injuries or venous obstruction or due to sex hormone stimulation while leiomyomas arise from myometrial smooth muscle cells [7, 8].

### Clinical presentation

Angioleiomyomas present in the reproductive age group. In most cases, angioleiomyomas in the extremities present as painful, subcutaneous masses. In contrast, cervical angioleiomyomas, though extremely rare, can manifest with symptoms similar to other cervical or uterine pathology like heavy menstrual bleeding, pelvic pain, dysmenorrhea and pressure symptoms. There are case reports of severe haemorrhage and disseminated intravascular coagulation in uterine angioleiomyomas [9]. Presentation like pseudo- Meig’s syndrome, coagulopathy and even rupture of uterus have also been reported [9–11]. Pain is mainly due to ischemia or vascular obstruction while abnormal bleeding is due to dysregulated angiogenesis and increase in growth receptors.

Histopathologically these tumors are of cavernous, venous or solid subtypes. Solid types have abundant smooth muscle bundles compactly surrounding the vascular channels. Cavernous type tumors have dilated vascular channels with scanty smooth muscle tissue component. Venous types have vascular channels with thick walls and less muscle tissue. These tumors may undergo hyaline or myxoid degeneration [2]. Malignant transformation of these tumors is extremely rare [12].

In the index case, the patient’s primary symptoms were chronic heavy menstrual bleeding and intermittent pelvic pain, both of which are common presentations for many gynecological disorders such as fibroids, adenomyosis, and endometriosis. This can make diagnosis challenging, as these symptoms are nonspecific and common in various benign conditions of the reproductive tract. Reproductive age, chronic heavy menstrual bleeding, pelvic pain, and the tumor’s vascular predominance predisposed the index patient to anaemia and hemorrhagic complications.

### Diagnostic imaging

Ultrasound is typically the first-line imaging modality for evaluating abnormal bleeding and pelvic masses. Angioleiomyomas typically appear as hypoechoic masses on ultrasound, similar to leiomyomas. However, MRI is more definitive, as the vascular component of these tumors can often be highlighted with hyper intensity on T2-weighted images.

### Histopathology

Histopathologically, angioleiomyomas are characterized by interlacing bundles of smooth muscle cells with numerous thick-walled blood vessels. The mass is usually well circumscribed or encapsulated in nature. There is no significant hypercellularity, nuclear atypia, increased mitosis or necrosis noted in this tumor.

Immunohistochemistry (IHC) often shows positivity for SMA, desmin, and sometimes vimentin and h-caldesmon, confirming smooth muscle origin [8].

Differential diagnoses for such a tumor include angiomyolipoma, endodermal stromal tumor, low grade perivascular epithelioid cell tumor and other vascular tumors. Histopathological study and IHC helps to differentiate these tumors.

### Treatment

The definitive treatment for cervical angioleiomyomas is surgical excision, either through hysterectomy or localized excision, depending on the size and location of the tumor and the patient’s reproductive preferences. In our case, complete excision via a vaginal approach successfully resolved the patient’s symptoms.

### Prognosis

The prognosis for cervical angioleiomyomas is excellent, with complete resection being curative. Recurrence is extremely rare, and there have been no reports of malignant transformation in these tumors [12]. Periodic clinical and imaging surveillance is recommended to confirm complete excision, monitor hematological recovery, and assess fertility outcomes.

### Clinical implication

This case highlights the critical importance of considering angioleiomyoma in the differential diagnosis of cervical masses, especially in reproductive – age women presenting with chronic heavy menstrual bleeding and pelvic pain. Although rare, angioleiomyoma can mimic common gynecological conditions like fibroids or adenomyosis, leading to potential misdiagnosis and delayed treatment. In this patient, advanced imaging with MRI revealed a hyperintense lesion suggestive of vascularity, prompting surgical excision and histological confirmation of angioleiomyoma. The tumor’s prominent vascular component explained the patient’s anaemia and heavy bleeding, and its benign nature allowed for conservative management with complete symptom resolution. IHC played a pivotal role in distinguishing this tumor from other vascular tumors or stromal neoplasms, reinforcing the need for thorough in distinguishing this tumor from other vascular or stromal neoplasms, reinforcing the need for thorough pathological evaluation. Clinicians should be aware that while angioleiomyomas are uncommon, their inclusion in diagnostic considerations can guide appropriate imaging, prevent complications, and support fertility-preserving treatment strategies.

## CONCLUSIONS

Cervical angioleiomyoma, though rare, should be considered in the differential diagnosis of young females presenting with persistent heavy menstrual bleeding and pelvic pain, especially when imaging reveals a well-circumscribed cervical mass with vascular components. Surgical excision offers curative treatment, with excellent prognosis and symptomatic relief.
